# Cancer Metabolism and Resistance to Cell Death: Novel Therapeutic Perspectives

**DOI:** 10.3390/biomedicines10081828

**Published:** 2022-07-29

**Authors:** Francesco Ciccarese

**Affiliations:** 1 Immunology and Molecular Oncology Unit, Veneto Institute of Oncology IOV-IRCCS, 35128 Padova, Italy; francesco.ciccarese@iov.veneto.it; 2 Department of Pathology and Laboratory Medicine, Weill Cornell Medicine, New York, NY 10065, USA

Deregulation of metabolism and resistance to cell death are two hallmarks of cancer [1]. A plethora of genetic and non-genetic processes tightly control catabolic and anabolic reactions in cancer cells, thus sustaining uncontrolled proliferation, survival in an adverse microenvironment, invasion, metastasis, and resistance to anticancer therapies.

Evasion of cell death is the leading cause of therapeutic failure, thus representing the main issue that oncologists must deal with. Although several mechanisms of cell death have been identified to tightly control cell viability, clonal evolution endows cancer cells with the ability to curb these mechanisms. Dysregulated cancer metabolism promotes resistance to cell death by activating pro-survival processes, including autophagy [2], or favoring immune escape by modulating the tumor microenvironment (TME) [3]. The pivotal role of metabolic reprogramming in the resistance of cancer cells to different types of cell death provides the rationale for anticancer strategies aimed at rewiring cancer cell metabolism.

Articles collected in the Special Issue “Cancer metabolism and resistance to cell death: novel therapeutic perspectives” highlight the tight connections between metabolic deregulation and survival of cancer cells in a harsh environment. 

The link between dysregulated metabolism and the resistance of cancer cells in an adverse microenvironment is a key point emerging from all the review articles published in this Special Issue. Surviving in an unfavorable milieu requires rapidity, evasiveness, and adaptation. Gundamaraju and colleagues discussed the rapid integration of external stimuli by G-protein-coupled receptors (GPCRs) and the role that endoplasmic reticulum (ER) stress and epithelial-to-mesenchymal transition (EMT) play in tumorigenesis, focusing on possible therapeutic targets [4]. Two processes are pivotal in the resistance of cancer cells in an adverse environment: (i) the unfolded protein response (UPR), which relieves the ER stress imposed by dysregulated cancer metabolism; and (ii) autophagy. Pharmacological targeting of these two pathways—with salubrinal to inhibit the dephosphorylation of eukaryotic translation initiation factor 2α (eIF2α) and with chloroquine or other drugs to inhibit autophagy—is a viable strategy to sensitize cancer cells to chemotherapy. Importantly, autophagy also controls, both positively and negatively, EMT, a pivotal process during invasion and metastasis.

Cruz-Bermúdez and colleagues focused on evasiveness as a key feature to surviving in a harsh environment [3]. The TME is populated by several non-cancer cell types, among which there are effectors of the immune system. Immune evasion is essential for cancer cell survival in the tumor milieu. Hypoxia downregulates major histocompatibility complex (MHC) expression while promoting, along with EMT, the expression of PD-L1, thus resulting in the resistance of cancer cells to immune elimination. Moreover, autophagy reduces the susceptibility of cancer cells to immune effectors and induces the degradation of granzyme derived from cytotoxic T-cells and NK cells. Several inhibitors of metabolic enzymes are in clinical trials in combination with immune checkpoint inhibitors. Pharmacological targeting of indoleamine 2,3-dioxygenase (IDO), arginase, lactate dehydrogenases (LDH), reactive oxygen species (ROS), adenosine, glucose and glutamine metabolism, and hypoxia-inducible factor 1α (HIF-1α) are all viable strategies to bypass resistance mechanisms put in place by cancer cells to evade immune control.

Genetic mutations in oncogenes and tumor suppressor genes play an important role in the adaptation of cancer cells to harsh conditions in the TME. Iessi and colleagues discussed the metabolic characteristics that distinguish normal from malignant cells, with a focus on possible therapeutic interventions [5]. Harsh conditions in the TME, in particular hypoxia, drive the metabolic switch of cancer cells from oxidative phosphorylation (OXPHOS) to glycolysis, thus leading to the acidification of the TME, the increased expression of proton pumps, and the selection of cancer cells highly resistant to cell death. Moreover, the acidic TME also impairs the cytotoxic activity of many chemotherapeutic drugs through their direct protonation-induced inactivation and HIF-1α-driven upregulation of drug efflux transporters. In this context, therapeutic approaches targeting proton pumps are a viable strategy to induce apoptosis of cancer cells. Interestingly, other cell death mechanisms, such as necroptosis and ferroptosis, are also linked to cancer metabolism, thus providing the rationale for targeting metabolic deregulation to kill apoptosis-resistant cancer cells.

If genetic mutations affect genes encoding for metabolic enzymes, a neomorphic activity could result, leading to the generation of new metabolites, known as oncometabolites, that are peculiar to cancer cells. Raimondi and colleagues discussed the role that one of these oncometabolites, 2-hydroxyglutarate (2-HG), plays in the biology of acute myeloid leukemia (AML) and its response to therapies [6]. Interestingly, 2-HG released from AML cells activates the NF-κB pathway in stromal cells of the bone marrow, thus creating a supportive niche that drives the proliferation and resistance of AML cells to chemotherapeutic agents. In this context, reducing 2-HG levels through the pharmacological inhibition of mutated isocitrate dehydrogenase (IDH) enzymes is an effective strategy, mostly when combined with chemotherapy or the BH3-mimetic drug venetoclax, to improve the outcome of AML patients. 

Two research articles published in the Special Issue highlight the anticancer efficacy of oxidative stress as a consequence of the pharmacological modulation of cancer metabolism. Ravera and colleagues demonstrated that 810 nm photobiomodulation (PBM) therapy impairs mitochondrial metabolism and decreases catalase activity, thus inducing ROS-mediated apoptosis in a model of head and neck squamous cell carcinoma [7].

Bierhals and colleagues showed that glycine uptake through the transporter GLYT1 is necessary to sustain the proliferation of colorectal and non-small-cell carcinoma rapidly proliferating cells [8]. Interestingly, glycine can be incorporated directly and indirectly (through one-carbon metabolism and the trans-sulphuration pathway) into glutathione, thus contributing to ROS scavenging. Indeed, the pharmacological inhibition of GLYT1 led to the sensitization of cancer cells to stress conditions, such as oxidative stress and ER stress.

In conclusion, the articles published in the Special Issue outlined the metabolic features that allow cancer cells to survive in an adverse environment, with a focus on therapeutic strategies that, impinging on cancer metabolism, could bypass the resistance of cancer cells to several types of cell death. The implementation of such strategies in clinical practice in the future could drastically change the outlook of relapsed/refractory cancer patients by turning the TME into an unlivable environment.
